# Study on the Function of the Inositol Polyphosphate Kinases Kcs1 and Vip1 of *Candida albicans* in Energy Metabolism

**DOI:** 10.3389/fmicb.2020.566069

**Published:** 2020-12-10

**Authors:** Xueling Peng, Qilin Yu, Yingzheng Liu, Tianyu Ma, Mingchun Li

**Affiliations:** Key Laboratory of Molecular Microbiology and Technology, Ministry of Education, Department of Microbiology, College of Life Sciences, Nankai University, Tianjin, China

**Keywords:** *Candida albicans*, glycolysis, mitochondria, lipid droplet, cell membrane permeability

## Abstract

In *Saccharomyces cerevisiae*, inositol polyphosphate kinase *KCS1* but not *VIP1* knockout is of great significance for maintaining cell viability, promoting glycolysis metabolism, and inducing mitochondrial damage. The functions of *Candida albicans* inositol polyphosphate kinases Kcs1 and Vip1 have not yet been studied. In this study, we found that the growth rate of *C. albicans vip1*Δ/Δ strain in glucose medium was reduced and the upregulation of glycolysis was accompanied by a decrease in mitochondrial activity, resulting in a large accumulation of lipid droplets, along with an increase in cell wall chitin and cell membrane permeability, eventually leading to cell death. Relieving intracellular glycolysis rate or increasing mitochondrial metabolism can reduce lipid droplet accumulation, causing a reduction in chitin content and cell membrane permeability. The growth activity and energy metabolism of the *vip1*Δ/Δ strains in a non-fermentable carbon source glycerol medium were not different from those of the wild-type strains, indicating that knocking out *VIP1* did not cause mitochondria damage. Moreover, *C. albicans KCS1* knockout did not affect cell activity and energy metabolism. Thus, in *C. albicans*, Vip1 is more important than Kcs1 in regulating cell viability and energy metabolism.

## Introduction

*Candida albicans* is a conditionally pathogenic fungus, and a comprehensive and in-depth understanding of the fungus helps us to treat and prevent the pathogen more effectively. Normal energy metabolism is conducive to maintaining the metabolic balance of the living body (Gan et al., 2010), and an in-depth exploration of the energy metabolism of *C. albicans* helps us to have a deep understanding of the pathogen.

In *C. albicans*, energy metabolism is closely related to its virulence such as hyphae development, cell wall synthesis, and drug resistance. *C. albicans* is a Crabtree-negative cell, which means that its growth is mainly dependent on mitochondrial oxidative phosphorylation, and damage to the mitochondrial respiratory chain will directly affect the viability of the cell (Duvenage et al., 2019). The anti-fluconazole mechanism of *C. albicans* can be attributed to the presence of its efflux pump system, and mitochondria play an important role in this process (Guo et al., 2017). Mutations in the mitochondrial respiratory chain complex will affect mitochondrial function; the cell wall, cell membrane, glycolysis, and glycosynthesis; and the reactive oxygen species (ROS) scavenging system of the cells (She et al., 2018). Hypha is the pathogenic state of *C. albicans*, but respiratory metabolism is not necessary for the transformation of *C. albicans* from yeast morphology to hyphae (Sun et al., 2019). Changing the expression of glycolysis-related genes will affect the transformation of *C. albicans*. Ace2, a transcription factor, promotes glycolysis and reduces cellular respiration. The knockout of the *ACE2* gene slowed the development of hyphae, suggesting that the increased glycolysis of *C. albicans* can promote hypha development (Mulhern et al., 2006). In addition, disrupting the energy balance can affect the virulence of *C. albicans*. For example, inhibiting the rate of glycolysis can reduce the formation of biofilm and the adhesion ability of the strain (Bonhomme et al., 2011). Glyceraldehyde-3-phosphate dehydrogenase (GAPDH), a glycolytic metabolic enzyme, also exists as a surface antigen in *C. albicans*, which can be used as a signal recognized by the immune system when *C. albicans* infects the human body (Gil-Navarro et al., 1997). In addition, more glycolysis-related metabolic enzymes in *C. albicans* are recognized as antigens in the plasma of some patients (Swoboda et al., 1993).

The mitochondria of *C. albicans* play an important role in normal growth and morphogenesis. Inhibition of respiration leads to loss of viability and cell wall rearrangements, which further promotes the phagocytosis of the strain by macrophages (Duvenage et al., 2019). The cell wall plays a key role in the viability of *C. albicans*, and it is a major determinant of virulence. Cell wall components and structure are the keys to recognition by the host immune system (Gow et al., 2007; Netea et al., 2008; McKenzie et al., 2010). Mitochondrial function is closely related to the maintenance of the *C. albicans* cell wall. Knockout of the regulatory protein Goa1 of mitochondrial complex I changes the cell wall structure, the strain becomes sensitive to cell wall damage reagents, and the ability of innate immune cells to recognize the strain is reduced (She et al., 2013). Knockout of the mitochondrial GTPase *Gem1* makes the strain sensitive to cell wall damage reagents and lack invasive growth (Koch et al., 2017). In summary, disrupting the balance of energy metabolism will seriously affect the viability and virulence of *C. albicans*.

The synthesis of inositol polyphosphate molecules IP7 and IP8 is catalyzed by Kcs1 and Vip1 in *Saccharomyces cerevisiae* and by inositol hexakisphosphate kinase (IP6K) and diphosphoinositol pentakisphosphate kinase (PPIP5Ks) in mammalian cells (Szijgyarto et al., 2011; Gu et al., 2017). However, the functions of Kcs1 and Vip1 in *C. albicans* have not been studied yet.

Inositol polyphosphate is a class of molecules containing high-energy bisphosphate groups. Compared with ATP, an energy molecule, one of its phosphate groups can be hydrolyzed more easily, thereby participating in the regulation of intracellular ATP levels (Szijgyarto et al., 2011; Gu et al., 2017).

There have been many reports about the participation of inositol polyphosphate molecules in energy metabolism (Szijgyarto et al., 2011). Knocking out the *IP6K* in mice will promote energy consumption in the body and reduce fat accumulation and body weight (Zhu et al., 2017). *IP6K1−/−* mice can avoid weight gain and insulin resistance caused by high-fat feeding, owing to the activation of the AKT pathway, therefore inhibition of IP6K1 can be used as a strategy for the treatment of obesity and diabetes (Chakraborty et al., 2010).

Regarding the regulation of IP6K1 in energy metabolism, Szijgyarto et al. (2011) published a classic article in 2011 that pointed out that knocking out the *KCS1* gene in *S. cerevisiae* will promote the rate of intracellular glycolysis and accelerate energy consumption. Inositol polyphosphate regulates ATP content by regulating glycolysis and mitochondria. It is worth mentioning that knocking out *KCS1* in *S. cerevisiae* can cause mitochondrial damage. The reasonable speculation for this is that mitochondrial damage is caused by gene knockout, to balance the energy metabolism of the body, cells will provide more energy by increasing the metabolic rate of glycolysis, thus increased carbon source consumption and reduced fat accumulation. A study in mice demonstrated that an increase in the rate of glycolysis was accompanied by energy consumption by the body, which in turn led to a decrease in fat content and weight loss (Wu et al., 2005). Knocking out *PPIP5K* in HCT116 cells can increase the rate of glycolysis and lead to excessive metabolism (Gu et al., 2017).

Preliminary research in our laboratory found that *KCS1* knockout did not affect *C. albicans* systemic infection in mice (Xiaoling et al., 2017); *VIP1* knockout strains infected the mice, and the mortality of the mice and the fungal burden in the kidneys of the mice were significantly reduced (Tianyu et al., 2020). That is, deletion of *VIP1* will affect the virulence and pathogenicity of *C. albicans*.

This study aims to explore the significance of *C. albicans* Kcs1 and Vip1 in cell growth and energy metabolism, including glycolysis, mitochondrial activity, and lipid droplet metabolism. Our results showed that knocking out the *C. albicans VIP1* gene will not damage the mitochondria but promote glycolysis. The excessive accumulation of lipid droplets in the *vip1*Δ/Δ strains is the cause of increased cell membrane permeability and cell death. This study shows that inositol polyphosphate kinase Vip1 of *C. albicans* is more important than Kcs1 in regulating energy metabolism and cell viability.

## Results

### Knockout of *VIP1* but Not *KCS1* Causes the Strain to Grow Slower and Lipid Droplets to Accumulate

*Saccharomyces cerevisiae KCS1* instead of *VIP1* knockout made the strain unable to survive in a non-fermentable carbon source medium, caused mitochondrial damage, and increased glycolysis metabolic rate (Szijgyarto et al., 2011). In this study, *C. albicans kcs1*Δ/Δ and *vip1*Δ/Δ strains (Supplementary Figure S1) were grown in a fermentable carbon source glucose or a non-fermentable carbon source glycerol medium to determine their status. Strains with abnormal glycolysis metabolism also grew abnormally in glucose medium, while strains with damaged mitochondria could not survive in glycerol medium (Robinson et al., 1992).

**FIGURE 1:**
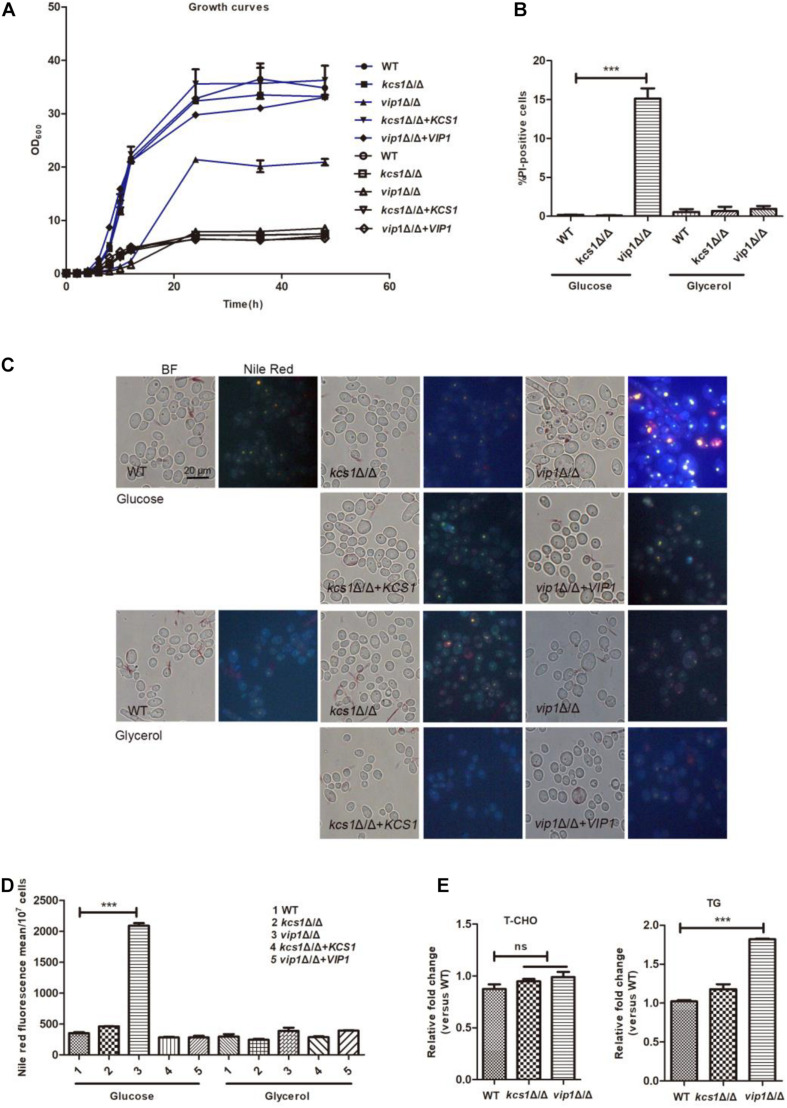
Phenotype of *C. albicans KCS1*, *VIP1* gene knockout. **(A)** Growth curve. Growth curves of wild-type (WT), *kcs1*Δ/Δ, *vip1*Δ/Δ, *kcs1*Δ/Δ + *KCS1*, and *vip1*Δ/Δ + *VIP1* strains in glucose (blue) or glycerol medium (black). **(B)** Measurement of cell membrane permeability (cell death). The strains were grown in glucose or glycerol medium, propidium iodide (PI) staining, and determined by flow cytometry (BD FACSCalibur, BD, United States). **(C,D)** Measurement of lipid droplets. The strains were grown in glucose or glycerol medium to stationary phase, stained with Nile red, and observed with fluorescence microscope (BX-53, Olympus, Japan) **(C)** or the fluorescence intensity was determined by microplate reader (Perkin Elmer, United States) **(D)**. **(E)** Determination of T-CHO and triglyceride (TG) content of WT, *kcs1*Δ/Δ, and *vip1*Δ/Δ strains in glucose medium. *P < 0.05.

In glucose medium, the growth rate and biomass of the *vip1*Δ/Δ strains were significantly lower than those of the wild-type (WT) (Figure 1A); the positive rate of propidium iodide (PI) staining (measured using flow cytometry) (Figure 1B and Supplementary Figure S2), the content of lipid droplets (stained with Nile red dye) (Figures 1C,D), and triglyceride (TG) [total cholesterol (T-CHO)] were significantly higher than those of the WT strains (Figure 1E).

The *vip1*Δ/Δ grew in glycerol medium, although the growth rate in the early stage of cultivation was slightly lower than that of the WT, when the strain grew to the stationary phase, the biomasses of the *vip1*Δ/Δ and the WT strains were not different (Figure 1A); the positive rates of PI staining and lipid droplet content of the *vip1*Δ/Δ under this culture condition were not different from those of the WT strains (Figures 1B–D).

*KCS1* knockout did not affect the growth of *C. albicans* in glucose or glycerol medium, and the accumulation of lipid droplets within the strain was also not different from that of the WT (Figures 1A–E).

The above results show that the knockout of *VIP1* instead of the *KCS1* gene affects the growth rate and lipid accumulation of *C. albicans* grown in glucose medium. The growth rate of *vip1*Δ/Δ grown in glycerol medium was not different from that of the WT strain. We speculate that the knockout of *VIP1* will not cause mitochondrial damage. The reason why the *vip1*Δ/Δ strain growth in glucose medium was reduced and lipid droplets accumulated may be due to abnormal glycolysis.

### Knocking Out *VIP1* Instead of *KCS1* Increased the Rate of Glycolysis

**FIGURE 2:**
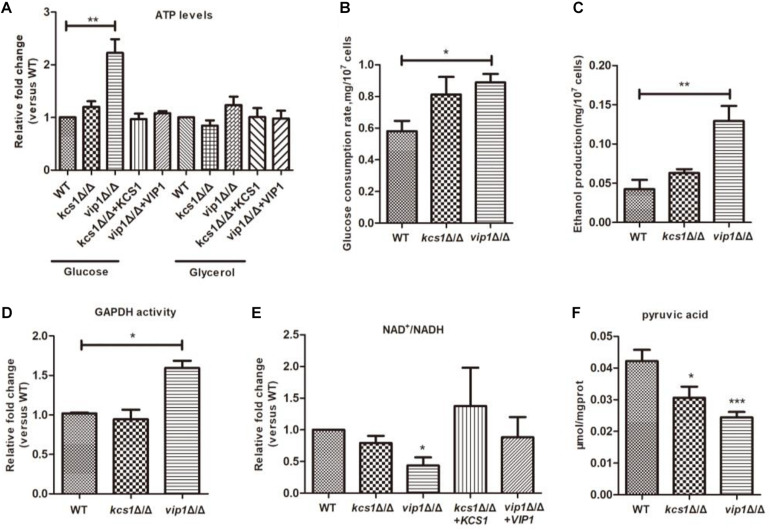
The effect of *KCS1*, *VIP1* gene knockout on glycolysis metabolism. **(A)** ATP content determination. Collected strains grown in glucose or glycerol medium to determine ATP content. **(B)** Glucose consumption rate. **(C)** Ethanol production. **(B,C)** Growing the strain in glucose medium, the mid-log phase strains were collected and centrifuged; the supernatant was used for the determination of glucose and ethanol content. **(D–F)** The strain was grown in glucose medium, then the cells were collected, glass beads were added and vortexed to break the strain, and the supernatant was collected after centrifugation to determine the activity of GADPH **(D)**, NAD^+^/NADH ratio **(E)**, and pyruvate content **(F)**. *P < 0.05.

ATP plays an important role in energy metabolism (Szijgyarto et al., 2011). Both glycolysis and the mitochondrial respiratory chain are sources of ATP production. When the *vip1*Δ/Δ was grown in glycerol medium, the ATP content of the strain was not different from that of the WT, but in glucose medium, the ATP content of the *vip1*Δ/Δ was significantly higher (Figure 2A) and ATP/ADP ratios were increased (Supplementary Figure S3). We speculate that the large amount of ATP in the *vip1*Δ/Δ strains was due to abnormal glycolysis (Lu J. et al., 2015).

The indicators related to glycolysis include glucose consumption rate, ethanol synthesis, and GAPDH activity. For the *vip1*Δ/Δ grown in glucose medium, these indicators of glycolysis were significantly higher than those of the WT strains (Figures 2B–D), indicating that the knockout of the *VIP1* will promote the rate of glycolysis.

Once the glycolysis rate is too high, nicotinamide adenine dinucleotide (NADH) will accumulate in the cytoplasm, thereby affecting the conversion efficiency of NAD^+^ to NADH, reducing the ratio of NAD^+^/NADH, and breaking the intracellular redox balance (Shimizu, 2018). For the *vip1*Δ/Δ strains grown in glucose medium, the intracellular NADH is nearly twice more than that of NAD^+^ (Figure 2E). This indicates that excessive glycolysis in the *vip1*Δ/Δ strains disrupts the energy balance.

Excessive intracellular NADH will affect the redox balance, at this time, pyruvate produced by glycolysis cannot be metabolized through the tricarboxylic acid (TCA) cycle but enters the fermentation pathway (McKnight, 2010; Lu J. et al., 2015; Chauhan et al., 2019; Fan and Sun, 2019). *Achatina fulica* infected by *Angiostrongylus cantonensis* can activate the anaerobic fermentative metabolism, affect oxidative metabolism, and cause pyruvic acid produced by glycolytic metabolism to enter the fermentation pathway to produce lactic acid, reducing pyruvic acid content (Tunholi-Alves et al., 2018). Our results showed that the content of pyruvate in the *vip1*Δ/Δ was significantly lower than that in the WT strains (Figure 2F). Excessive NADH will inhibit the activity of the enzymes in the mitochondrial TCA cycle, thus pyruvate does not enter the TCA cycle but produces ethanol through the fermentation route (Gerhart-Hines et al., 2007; Ikon and Ryan, 2017).

The above results indicate that *C. albicans VIP1* knockout promotes intracellular glycolysis, but *KCS1* knockout does not affect glycolysis (Figures 2A–F).

### *vip1*Δ/Δ Grown in Glucose Instead of Glycerol Has Reduced Mitochondrial Activity

A non-fermentable carbon source is a carbon source for mitochondrial respiratory metabolism (Li D. et al., 2016). Cells with damaged mitochondria cannot grow in a non-fermentable carbon source medium (Robinson et al., 1992; Saibabu et al., 2017). The growth status of *C. albicans kcs1*Δ/Δ and *vip1*Δ/Δ in non-fermentable carbon source glycerol medium was not different from that of the WT strain. We speculate that neither *KCS1* nor *VIP1* knockout will damage mitochondria.

**FIGURE 3:**
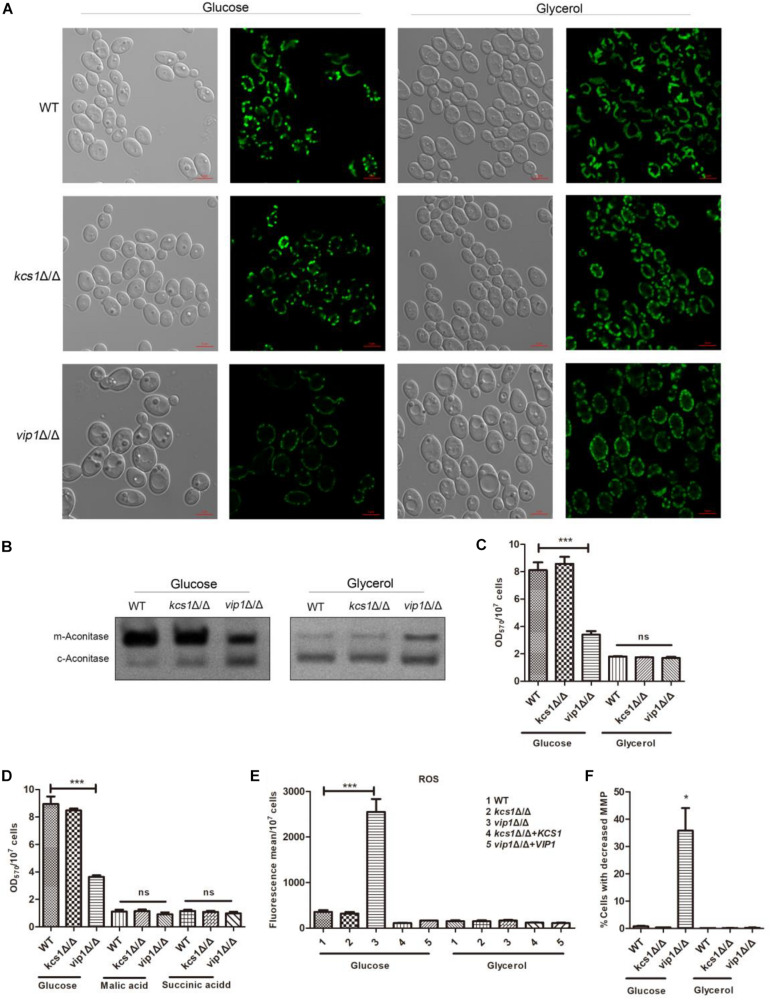
The effect of *KCS1*, *VIP1* knockout on mitochondrial activity. **(A)** Observation of mitochondrial morphology. WT-Csp37-GFP, *kcs1*Δ/Δ-Csp37-GFP, and *vip1*Δ/Δ-Csp37-GFP strains were grown in glucose or glycerol medium. The mitochondrial morphology was observed by confocal laser scanning microscopy (CLSM) images. **(B)** The mitochondrial aconitase activity was measured using the in-gel enzyme activity assay (IGA) method. The strains were grown in glucose or glycerol medium. Strains were collected and glass beads were added, vortexed to break the strain, and then centrifuged to collect the supernatant for aconitase activity determination. **(C)** 3-(4,5-Dimethylthiazol-2-yl)-2,5-diphenyltetrazolium bromide (MTT) assay for measuring mitochondrial succinate dehydrogenase activity of strains grown in glucose or glycerol medium. **(D)** MTT assay for measuring mitochondrial succinate dehydrogenase activity of strains grown in malic acid or succinic acid medium. **(E)** Reactive oxygen species (ROS) determination. The strains were grown in glucose or glycerol medium. The ROS content of the strains was stained with 2,7-dichlorodihydro-fluorescein diacetate (DCFH-DH) dye, and the fluorescence content was measured with a microplate reader (Perkin Elmer, United States). **(F)** Determination of mitochondrial membrane potential. The strains were grown in glucose or glycerol medium. After collecting the cells, they were stained with JC-1 dye [0.5 mg/ml, dissolved in dimethyl sulfoxide (DMSO), Sigma, United States] and the mitochondrial membrane potential was measured by flow cytometry (DB FACSCalibur, BD, United States). *P < 0.05.

Mitochondrial morphology is closely related to its function (Roy et al., 2019), and the dynamic balance between mitochondrial fusion and fission is important for its function (Kobayashi et al., 2002). We used the Csp37-GFP fusion protein to characterize the mitochondrial morphology of *C. albicans* (Dong et al., 2015). The mitochondria of *kcs1*Δ/Δ and *vip1*Δ/Δ strains grown in glycerol medium did not show swelling or shrinkage (Sun et al., 2019) (Figure 3A).

Succinate dehydrogenase is a key enzyme at the junction between the electron transport chain and the TCA cycle; mitochondrial aconitase (*m*-aconitase) can be used as an indicator of whether the TCA cycle is working properly. These enzyme activity parameters can be used as a reference to determine whether the mitochondrial function is normal. We used the in-gel activity assay and 3-(4,5-dimethylthiazol-2-yl)-2,5-diphenyltetrazolium bromide (MTT) method to measure aconitase (including mitochondrial aconitase and cytoplasm aconitase) and succinate dehydrogenase activities (Pereira et al., 2019).

The mitochondrial aconitase and succinate dehydrogenase activities of the *kcs1*Δ/Δ grown in glycerol medium were not affected; the mitochondrial succinate dehydrogenase activity of *vip1*Δ/Δ grown in glycerol medium was not different from that of the WT strains (Figure 3C), while its mitochondrial aconitase activity was higher (Figure 3B).

Cells with damaged respiratory chain cannot grow in succinate or malic acid medium (Overkamp et al., 2000; Vahsen et al., 2004). MTT results showed that the *kcs1*Δ/Δ and *vip1*Δ/Δ strains grown in the medium with malic acid or succinate acid as the sole carbon source had no difference in mitochondrial activity from the WT strain (Figure 3D); this result was consistent with the results of the culture assay in glycerol medium.

The reduction in mitochondrial oxidative phosphorylation efficiency will result in a large amount of ROS (Duvenage et al., 2019). The ROS content of *kcs1*Δ/Δ and *vip1*Δ/Δ strains grown in glycerol medium was not different from that of the WT strains (Figure 3E). In addition, the mitochondrial membrane potential (MMP) of *kcs1*Δ/Δ and *vip1*Δ/Δ strains was also unaffected (Figure 3F).

The above results indicate that the knockout of *KCS1* or *VIP1* will not affect the mitochondrial activity of *C. albicans* grown in glycerol medium; that is, the knockout of *KCS1* or *VIP1* will not cause mitochondrial damage.

*VIP1* knockout will not cause mitochondrial damage, but it does not mean that the mitochondrial function of the *vip1*Δ/Δ strain has always been in a normal state. Unlike the Crabtree-positive cell of *S. cerevisiae*, *C. albicans* is a Crabtree-negative cell, which means that even if the carbon source in the medium is glucose, as long as oxygen is present, *C. albicans* still produces ATP mainly through respiratory metabolism. According to this prompt, *vip1*Δ/Δ grows abnormally in glucose medium, in addition to the increase in the rate of glycolysis; so, will the mitochondria of the strain be affected?

The mitochondria-related metabolic indexes of the *vip1*Δ/Δ strains grown in glucose medium were measured, and the results were as follows: (1) there were no abnormalities such as swelling or shrinkage in the mitochondrial morphology (Figure 3A); (2) mitochondrial aconitase and succinate dehydrogenase activities were significantly lower than those of the WT strain (Figures 3B,C); (3) ROS content increased (Figure 3E); and (4) MMP decreased (Figure 3F). That is, the mitochondrial activity of *C. albicans vip1*Δ/Δ strain grown in glucose medium was reduced.

In summary, the results showed that *KCS1* and *VIP1* knockout does not affect the mitochondrial activity of *C. albicans* grown in glycerol medium; therefore, *KCS1* and *VIP1* knockout would not cause mitochondrial damage. *KCS1* knockout does not affect the mitochondrial function of the strain when grown in glucose medium, but the *VIP1* knockout strain reduced mitochondrial function.

### Increased Glycolysis Rate Accompanied by the Decreased Mitochondrial Function Will Cause Lipid Droplet Accumulation in the *vip1Δ/Δ*

When the *C. albicans vip1*Δ/Δ strain was grown in glucose medium, the glycolysis of the strain was upregulated, mitochondrial activity was reduced, and lipid droplets accumulated. In this regard, we question whether the accumulation of lipid droplets in *vip1*Δ/Δ is caused by abnormal glycolysis and mitochondrial metabolism (Liu et al., 2015).

**FIGURE 4:**
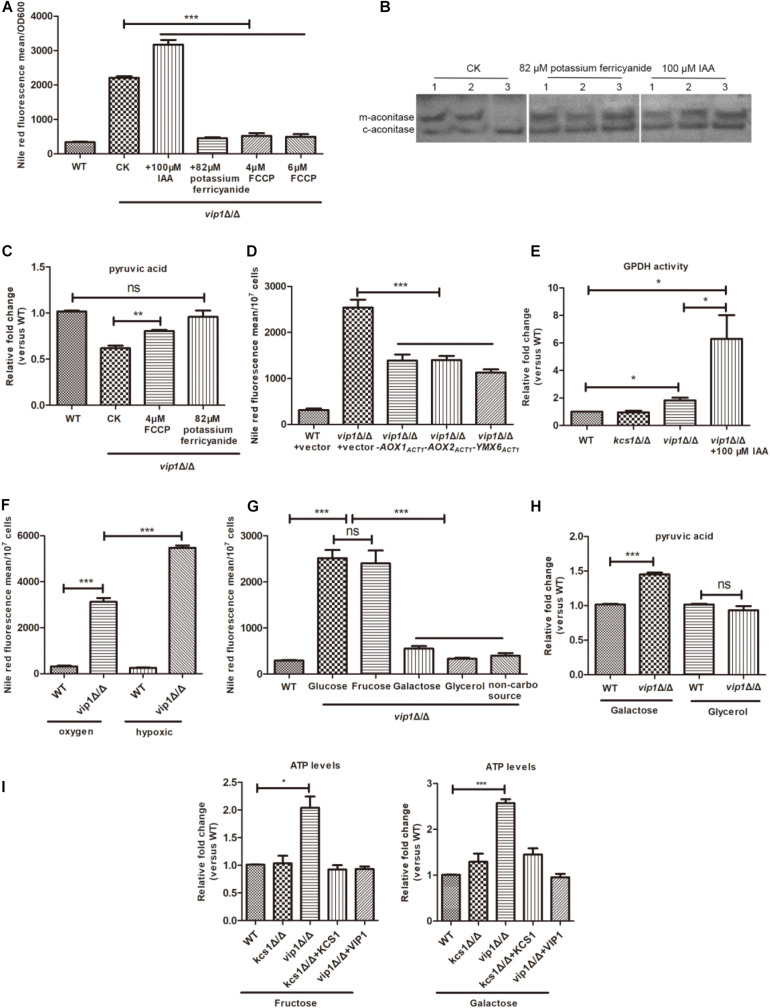
An increase in the rate of glycolysis in *vip1*Δ/Δ is accompanied by a decrease in mitochondrial activity. **(A)** Determination of lipid droplet content. The strain was grown in glucose medium or in glucose medium supplemented with 100 μM iodoacetic acid (IAA), 82 μM potassium ferricyanide, and 4 or 6 μM carbonyl cyanide 4-(trifluoromethoxy) phenylhydrazone (FCCP), and strains were collected that were grown to stationary phase and the lipid droplet content was measured. WT: wild-type strains were grown in glucose medium; CK: *vip1*Δ/Δ strains were grown in glucose medium without drug addition. **(B)** In-gel enzyme activity assays measure mitochondrial aconitase activity. (1) WT, (2) *kcs1*Δ/Δ, (3) *vip1*Δ/Δ. The strain was grown in glucose medium, or 82 μM potassium ferricyanide or 100 μM IAA was added to the glucose medium, and the aconitase activity was measured by isogeometric analysis (IGA) method. **(C)** Determination of pyruvate content. The strain was grown in glucose medium, or in glucose medium supplemented with 4 μM FCCP or 82 μM potassium ferricyanide, and the pyruvate content was measured. WT, wild-type strains were grown in glucose medium; CK, *vip1*Δ/Δ strains were grown in glucose medium without drug addition. **(D)** Determination of lipid droplet content. Determination of lipid droplet content of strains grown in glucose medium. **(E)** Glycerol-3-phosphate dehydrogenase (GPDH) activity. The strain was grown in glucose medium or glucose medium supplemented with 100 μM IAA, and GPDH activity was measured. **(F)** Determination of lipid droplet content under hypoxic conditions. The strain was grown in aerobic or hypoxic conditions, and the lipid droplet content was determined. **(G)** Determination of lipid droplet content in strains grown in different carbon sources. WT strains grown in glucose medium. **(H)** Determination of pyruvate content. The pyruvate content of strains in galactose or glycerol medium was determined. **(I)** Determination of ATP content of strains grown in fructose or galactose medium. *P < 0.05.

Excessively high glycolysis rate will cause the accumulation of NADH, while NAD^+^ and NADH in normal growing cells have been in a dynamic transition to maintain intracellular balance. When NADH accumulates in excess, it inhibits the enzyme of the mitochondrial TCA cycle, causing a reduction in mitochondrial activity (Liu et al., 2006; He et al., 2013). As an electron acceptor, potassium ferricyanide can promote the conversion of NADH to NAD^+^ (Kulikova, 2005; Wang et al., 2016). When potassium ferricyanide is added to the glucose medium, it can be taken up by cells and enter the cytoplasm but cannot enter the mitochondria; it then consumes NADH in the cytoplasm as an electron acceptor (Martinus et al., 1993; Mazumder et al., 2013), and the content of lipid droplets in the *vip1*Δ/Δ decreases (Figure 4A). Excessive NADH was consumed by potassium ferricyanide, which relieved the metabolic pressure in the cytoplasm and promoted the conversion of metabolic flow from pyruvate to the fermentation pathway to mitochondrial metabolism. At this time, the *vip1*Δ/Δ strain mitochondrial aconitase enzyme activity (Figure 4B) and intracellular pyruvate content increased (Figure 4C).

Carbonyl cyanide 4-(trifluoromethoxy) phenylhydrazone (FCCP) is a mitochondrial respiratory chain uncoupling agent; it promotes the oxidation of NADH from the cytoplasm by mitochondria and generates heat energy through the electron transport chain instead of ATP (Zhou et al., 2003). When the *vip1*Δ/Δ strains were grown in glucose medium supplemented with FCCP, the lipid droplet content of the *vip1*Δ/Δ strains decreased significantly (Figure 4A) with an increase in pyruvate content (Figure 4C).

*Candida albicans* mitochondria have both oxidative phosphorylation and alternative oxidase pathways. Aox1 and Aox2 are two proteins located on the alternative respiration pathway. They encode alternative oxidases and metabolize NADH in the mitochondria; Ymx6 is an NADH dehydrogenase located on the intermembrane space side of the mitochondria, which is responsible for mediating the transfer of electrons from cytosolic NADH to coenzyme Q (CoQ) (Overkamp et al., 2000; Gomes et al., 2013). Therefore, we suspect that the overexpression of NADH dehydrogenase in the mitochondria can increase mitochondrial activity to reduce the pressure from NADH. We grew *vip1*Δ/Δ + *PACT1-AOX1*, *vip1*Δ/Δ + *PACT1-AOX2*, and *vip1*Δ/Δ + *PACT1-YMX6* strains in glucose medium and measured their lipid droplet content, which was significantly lower for the *vip1*Δ/Δ strains (Figure 4D). This indicates that the consumption of NADH accumulated in the *vip1*Δ/Δ strains can reduce the metabolic pressure in the cells, improve mitochondrial activity (Supplementary Figure S4), and reduce the formation of lipid droplets.

The formation of lipid droplets is a means of compensation for the *vip1*Δ/Δ to balance the redox imbalance caused by NADH accumulation due to excessive glycolysis and reduced mitochondrial activity. Glycerol-3-phosphate dehydrogenase (GPDH) in the cytoplasm is a key enzyme that promotes the formation of lipid droplets. This enzyme uses glycerol-3-phosphate as a substrate and NADH as an electron donor, which in turn produces glycerol or TG and further forms lipid droplets. The *vip1*Δ/Δ grown in glucose medium had nearly twice the GPDH activity in the cytoplasm than the that in the WT strain (Figure 4E).

Iodoacetic acid (IAA) is a glycolysis inhibitor, and it promotes the conversion of glyceraldehyde-3-phosphate to dihydroxyacetone phosphate (DHAP) by inhibiting GAPDH. At this time, the metabolism from glyceraldehyde-3-phosphate to 1,3-diphosphoglycerate in glycolysis is weakened, and DHAP is further catalyzed by GPDH to synthesize glycerol-3-phosphate, which is the precursor of TG (Peña et al., 2015). Adding 100 μM IAA to the glucose medium increased the activity of the GPDH (Figure 4E), which promoted the formation of lipid droplets in the *vip1*Δ/Δ strains (Figure 4A).

IAA inhibits GAPDH activity during glycolysis, thereby alleviating glycolysis pressure in the *vip1*Δ/Δ strains; at the same time, mitochondrial activity was also increased (Figure 4B). The inhibition of glycolysis is accompanied by an increase in mitochondrial activity, which is called the Warburg-reversing effect (Lu C.L. et al., 2015). At the same time, it also shows that the knockout of the *VIP1* will not cause mitochondrial damage. The *vip1*Δ/Δ strains grown in glucose medium have a mitochondrial activity that decreases with increasing glycolysis and increases with decreasing glycolysis rate.

Under hypoxic conditions, the rate of glycolysis can be further increased with mitochondrial function inhibition (Suzuki et al., 2014). The lipid droplets of the *vip1*Δ/Δ strains under hypoxic conditions were significantly higher than when the strains were incubated under aerobic conditions (Figure 4F), and MTT experiments showed that the *vip1*Δ/Δ strains under hypoxic conditions had further mitochondrial activity suppression (Supplementary Figure S5). That is, an excessively high glycolysis rate is accompanied by a decrease in mitochondrial function, which is responsible for the accumulation of lipid droplets in *vip1*Δ/Δ strains.

When the *vip1*Δ/Δ strain grows in a medium with glucose as the sole carbon source, the phenomenon of excessive glycolysis accompanied by a decrease in mitochondrial activity is called the Warburg effect. However, once glucose is replaced with galactose, the Warburg effect is alleviated to some extent (Grimm et al., 2017).

By replacing the fermentable-type carbon source glucose with galactose, glycerol, or basic amino acid without carbon source culture (Ene et al., 2015), the lipid droplet content of the *vip1*Δ/Δ reduced significantly (Figure 4G). At this time, the energy produced by the strain was mainly through mitochondrial respiration, and the pyruvate content increased or was not different from that of the WT (Figure 4H). When glucose was replaced with a fermentable carbon source fructose, the content of lipid droplets in the *vip1*Δ/Δ increased significantly (Figure 4G). We measured the ATP content when fructose or galactose was used as the carbon source. As in the glucose medium, the ATP content of *vip1*Δ/Δ strains grown in fructose medium increased significantly. The *vip1*Δ/Δ strains grown in galactose medium also had significantly higher ATP content than that in WT (Figure 4I). Galactose is a carbon source that needs to be metabolized by mitochondria, but unlike glycerol, galactose must first undergo glycolysis and metabolism before it can enter the mitochondria for further use. Strains that use galactose as a carbon source have enhanced mitochondrial activity, but glucose and galactose metabolism yields equivalent amounts of ATP (Grimm et al., 2017). From this, we can be sure that the high glycolysis of the strain caused by knockout of the *VIP1* was accompanied by a decrease in mitochondrial function, which was responsible for the accumulation of the lipid droplets.

In summary, we found that knockout of the *C. albicans VIP1* gene does not cause mitochondrial damage, thus the growth and energy metabolism of the *vip1*Δ/Δ in a non-fermentable carbon source medium are not different from those of the WT strains, but the knockout of *VIP1* will promote glycolysis and induce the accumulation of lipid droplets when the strain grows in glucose medium.

### Excessive Accumulation of Lipid Droplets Leads to Increased Membrane Permeability of *vip1*Δ/Δ

**FIGURE 5:**
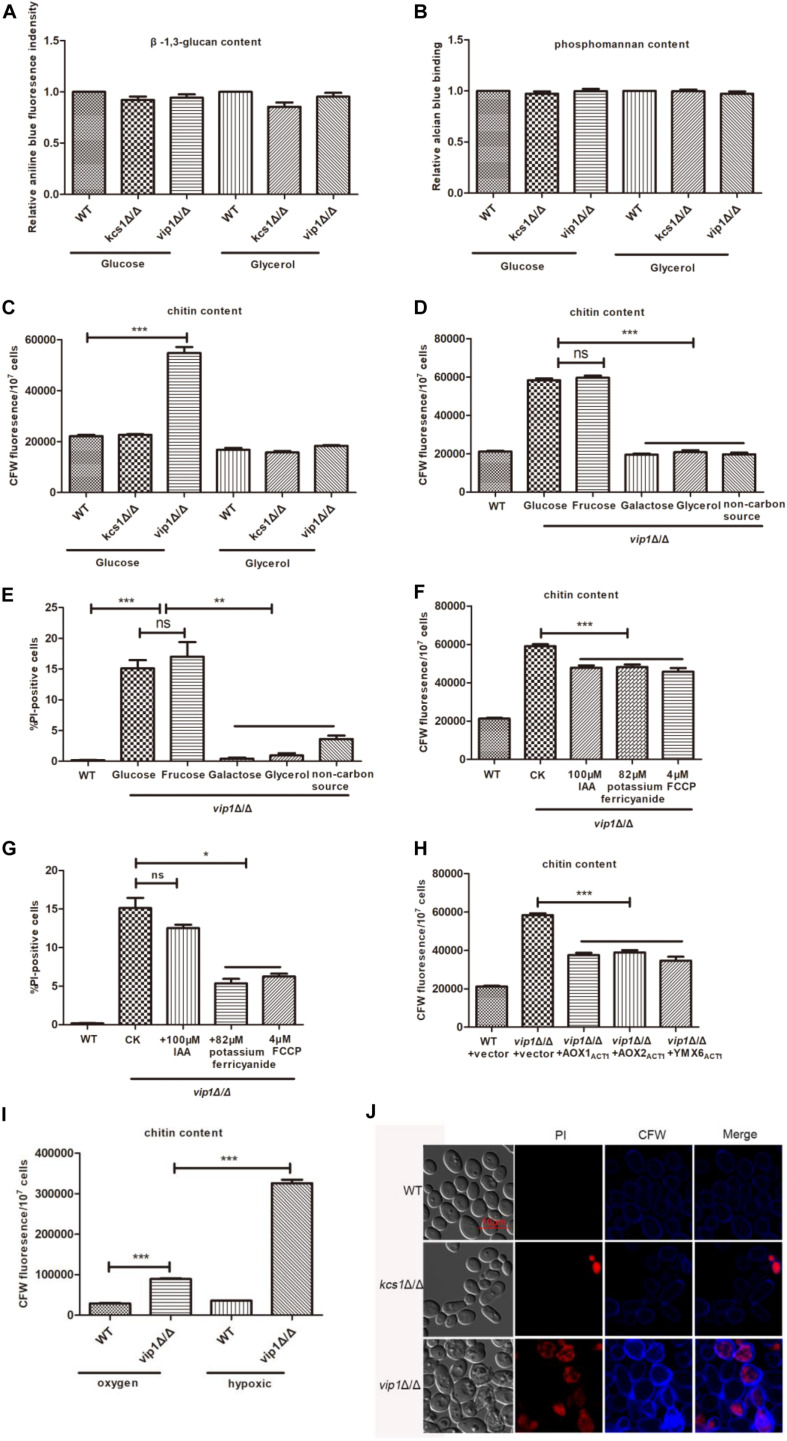
The accumulation of lipid droplets in *vip1*Δ/Δ strains promotes chitin synthesis and increased cell membrane permeability. **(A–C)** Determine the content of β-1,3-glucan, phosphomannan, and chitin in the cell wall. The strain was grown in a medium with glucose or glycerol as the sole carbon source, and the cell wall content was determined. **(D,E)** Determination of chitin content and cell membrane permeability (cell death) of strains under different carbon source culture conditions. The strain was grown in a medium with glucose, fructose, galactose, glycerol, or no carbon source (amino acid) as the carbon source. Calcofluor white (CFW) staining was used to determine the chitin content, and propidium iodide (PI) staining was used to determine the cell membrane permeability. Wild-type (WT) strains were grown in glucose medium. **(F)** Determination of chitin content. **(G)** Determination of cell membrane permeability (cell death). **(F,G)** *vip1*Δ/Δ strains were grown in glucose or glucose medium supplemented with 100 μM iodoacetic acid (IAA), 82 μM potassium ferricyanide, or 4 μM carbonyl cyanide 4-(trifluoromethoxy) phenylhydrazone (FCCP). WT strains were grown in glucose medium. **(H)** The strain was grown in glucose medium to determine the chitin content. **(I)** Determination of chitin content of strains under hypoxic conditions. The strains were grown under aerobic or hypoxic conditions, and CFW staining was used to determine chitin. **(J)** Strains were grown in glucose medium, collected and washed with PBS three times, and CFW and PI stained, then fluorescence observation was performed using confocal laser scanning microscopy (CLSM) images. *P < 0.05.

The *vip1*Δ/Δ grown in glucose medium accumulated a large amount of lipid droplets, and the cell membrane permeability increased. The cell wall is the part that is tightly connected to the plasma membrane, and it contains three main components: β-1,3-glucan, phosphomannan, and chitin. The content of β-1,3-glucan and phosphomannan in the *vip1*Δ/Δ grown in glucose medium was not different from that of the WT strains (Figures 5A,B), but the chitin content increased significantly (Figure 5C). The *vip1*Δ/Δ grown in glycerol medium showed no difference in cell wall composition from the WT (Figures 5A–C). After replacing the fermentable carbon source glucose with fructose, the chitin content of the *vip1*Δ/Δ strains increased; after replacing the non-fermentable carbon source glycerol with galactose, glycerol, or a non-carbon source (amino acid), the chitin content of *vip1*Δ/Δ was not different from that of the WT strain (Figure 5D). PI staining was used to measure the permeability of the plasma membrane. The *vip1*Δ/Δ grown in fermentable carbon source medium had increased cell membrane permeability; once the carbon source was replaced with a non-fermentable type, the cell membrane permeability decreased (Figure 5E). Together with the previous experimental results, we found that in the *vip1*Δ/Δ strains, chitin content and cell membrane permeability were positively correlated with the accumulation of lipid droplets.

To further verify this result, we added 82 μM potassium ferricyanide or 4 μM FCCP to the glucose medium and determined the chitin content and cell membrane permeability of the *vip1*Δ/Δ strain under this growth condition. The results showed that the chitin content and cell membrane permeability of *vip1*Δ/Δ reduced significantly (Figures 5F,G). The *vip1*Δ/Δ + *PACT1-AOX1*, *vip1*Δ/Δ + *PACT1-AOX2*, and *vip1*Δ/Δ + *PACT1-YMX6* strains grown in glucose medium had significantly lower chitin content than that of the *vip1*Δ/Δ (Figure 5H). In addition, the *vip1*Δ/Δ under hypoxic conditions had a significantly higher chitin content than that of the strain during aerobic status (Figure 5I). The chitin content of the *vip1*Δ/Δ grown in glucose medium supplemented with IAA was lower than when the inhibitor was not added to the medium, and there was no difference in cell membrane permeability between the two culture conditions (Figures 5F,G).

The accumulation of lipid droplets in the *vip1*Δ/Δ was accompanied by an increase in chitin and cell membrane permeability, and the cell membrane is closely linked to the cell wall. We posed a question: Is there a relationship between cell wall thickening and increased cell membrane permeability? We collected the strains grown in glucose medium, chitin content was determined using calcofluor white (CFW) staining, and PI staining was used to characterize cell membrane permeability. After microscopic observation, it was found that cells positive for PI staining in the *vip1*Δ/Δ were also darker in chitin staining (see the arrow in Figure 5J); conversely, cells with deep chitin staining were not necessarily PI-positive (Figure 5J). These results indicate that the thickening of the cell wall is responsible for the increased permeability of the *vip1*Δ/Δ strain membrane.

In summary, chitin content and cell membrane permeability in the *vip1*Δ/Δ strain are positively correlated with the accumulation of lipid droplets.

## Discussion

### Knockout of *Candida albicans VIP1* Instead of *KCS1* Affects Energy Metabolism

Knocking out *KCS1* instead of *VIP1* in *S. cerevisiae* promotes glycolysis rate and induces mitochondrial damage (Szijgyarto et al., 2011). In mice, *IP6K1* is the homolog of *KCS1* in *S. cerevisiae*, and knockout of this gene can also promote glycolysis and reduce lipid droplet content (Wu et al., 2005; Chakraborty et al., 2010; Lev et al., 2015; Zhu et al., 2017). However, the *KCS1* gene knockout did not affect the growth and energy balance of *C. albicans*; the knockout of *VIP1* promoted glycolytic metabolism rate, but the mitochondria were not damaged. This phenomenon is different from previous findings in *S. cerevisiae* and *Cryptococcus neoformans* (Lev et al., 2015). In *C. neoformans*, *KCS1* knockout resulted in cell wall integrity defect, and the growth of *kcs1*Δ in alternative carbon sources such as glycerol medium was significantly weakened; knockout of the *ASP1*/*VIP1* did not affect the virulence of the strain and had no effect on cellular function (Li C. et al., 2016; Lev et al., 2019). In yeasts, Kcs1 phosphorylates IP_5_ into 5-PP-IP_4_ and IP_6_ into 5-PP-IP_5_ (IP_7_); Vip1 phosphorylates IP_6_ into IP_7_ isomer 1-PP-IP_5_. (PP)_2_-IP_4_ (IP_8_) is generated by either Kcs1-mediated phosphorylation of 1-PP-IP_5_ or Vip1-mediated phosphorylation of 5-PP-IP_5_ (Banfic et al., 2016). But in *C. neoformans*, Kcs1 mediates the synthesis of PP-IP_5_/IP_7_, and Asp1/Vip1 further synthesize PP_2_-IP_4_/IP_8_ (Lev et al., 2019). In *C. albicans*, how Kcs1 and Vip1 act in the synthesis of inositol polyphosphate have not yet been studied, and this will be the direction of our later research. For *C. albicans*, Vip1 rather than Kcs1 is more important in regulating cell growth and energy metabolism.

For *C. albicans vip1*Δ/Δ strains grown in glucose medium, the increase in glycolysis was accompanied by a decrease in mitochondrial activity, thus leading to accumulation of lipid droplets; the relief of glycolysis was accompanied by an increase in mitochondrial activity, which is called the Warburg-reversing effect, and led to a reduction in the content of lipid droplets. This metabolic state reminds us of cancer cells. Cancer cells are dominated by glycolysis; even if the mitochondria function normally, they are usually in a low activity state. In future research, the *vip1*Δ/Δ of *C. albicans* can be used as a model strain to understand the physiological and metabolic status of cancer cells and provide a reference for future cancer research.

The *S. cerevisiae kcs1*Δ strain grown in glucose medium also exhibited an upregulation of glycolysis rate and a decrease in mitochondrial activity, but does this strain also have the same situation as the accumulation of lipid droplets in *C. albicans vip1*Δ/Δ strain? There is a very important parameter worthy of our attention, namely: the content of NADH. The NAD^+^ content in the *S. cerevisiae kcs1*Δ strain was significantly higher than that of NADH (Szijgyarto et al., 2011), but in the *C. albicans vip1*Δ/Δ strain, this is exactly the opposite (Figure 2E). The addition of potassium ferricyanide or FCCP (Figure 4A) or overexpression of NADH dehydrogenases such as Aox1, Aox2, or Ymx6 in the *vip1*Δ/Δ (Figure 4D) can promote the metabolism of NADH, increase mitochondrial activity (Supplementary Figure S4), and reduce the accumulation of lipid droplets. In addition, it was previously proposed that the difference between *S. cerevisiae* and *C. albicans* as Crabtree-positive and Crabtree-negative cells, respectively, is the difference in NADH metabolism between the two (Sakihama et al., 2019). *S. cerevisiae* is a Crabtree-positive cell; even under aerobic conditions, it uses glucose as a carbon source to produce ethanol instead of mitochondrial respiratory metabolism. The metabolic mechanism of glucose repression circuits in *S. cerevisiae* is currently not found in *C. albicans*. This metabolic mechanism can be expressed as: when glucose is used as the carbon source, the rate of glycolysis is increased, and the TCA cycle metabolism is inhibited. However, unlike it, Crabtree-negative cells, such as *C. albicans*, lack these glucose repression circuits in their cells, and once aerobic, they will mainly rely on respiratory metabolism to produce energy (Tao et al., 2017). It is precisely because of this characteristic of *C. albicans* that when the glycolysis rate of the *vip1*Δ/Δ strain is too high and the mitochondrial activity is reduced, the cells at this time are in a state of abnormal metabolism, and to balance intracellular metabolism, the *vip1*Δ/Δ strains produce lipid droplets.

Taking the *vip1*Δ/Δ strain of *C. albicans* as a model, we further understand the metabolic differences between this pathogenic fungus and the model organism of *S. cerevisiae* and further provide a reference for the treatment of this pathogen.

### The Accumulation of Lipid Droplets in *vip1*Δ/Δ Accompanies Increased Chitin Content and Membrane Permeability

The chitin content and cell membrane permeability in *vip1*Δ/Δ are positively correlated with the content of lipid droplets (Supplementary Figure S6). When the *vip1*Δ/Δ strains were grown in a non-fermentable carbon source medium, or when strain mitochondrial function is promoted by FCCP, the lipid droplet content, chitin, and cell membrane permeability of the *vip1*Δ/Δ strains are reduced to a certain extent. Together with the results shown in Figure 5J, we speculate that the cell membrane damage of *vip1*Δ/Δ strains is caused by the increase in chitin in the cell wall.

In view of the above results, we propose the following conjectures, and further results need more evidence to prove that: *vip1*Δ/Δ accumulates a large number of lipid droplets, which leads to an increase in intracellular osmotic pressure (cell swelling). To prevent the cells from swelling and rupturing, the cells respond to this pressure by thickening the cell wall (Udom et al., 2019). *C. albicans* chitin rather than β-1,3-glucan or phosphomannan is a component located in the inner layer of the cell wall, which is closely attached to the cell membrane (Peña et al., 2015), and the thickened cell wall stretches the cell membrane to increase the permeability of the cell membrane and cause cell death (Supplementary Figure S7).

In conclusion, in the *C. albicans vip1*Δ/Δ strain, lipid droplet accumulation is accompanied by the synthesis of chitin and an increase in cell membrane permeability. *VIP1* knockout promotes cell death, which provides ideas for the treatment of *C. albicans* pathogenicity.

## In Summary

This study demonstrates for the first time that inositol polyphosphate kinase Vip1 is more important than Kcs1 in regulating cell viability and energy metabolism of *C. albicans*. *KCS1* knockout does not affect *C. albicans* growth and energy metabolism (glycolysis, mitochondria, and lipid droplet metabolism). *VIP1* knockout does not cause mitochondrial damage, and the growth and energy metabolism of the *vip1*Δ/Δ strains grown in non-fermentable carbon source medium are not different from those of the WT; knockout of this gene will increase the rate of glycolysis, and *vip1*Δ/Δ strains grown in glucose medium have reduced mitochondrial activity and accumulated lipid droplets. Relieving the glycolysis rate of the *vip1*Δ/Δ strains or promoting mitochondrial activity can reduce the lipid droplet content. At the same time, chitin content and cell membrane permeability are positively correlated with the increase or decrease of lipid droplet content in the *vip1*Δ/Δ strains.

## Materials and Methods

### *Candida albicans* Strains and Plasmids

The primers used to construct strains and plasmids in this study are listed in Supplementary Table S1; all *C. albicans* strains used in this study are listed in Supplementary Table S2. *C. albicans* WT strain is BWP17 (Zhang et al., 2018), and the gene deletion or tagging was performed on these strains as previously described (Yu et al., 2014a; Knafler et al., 2019). Briefly, the gene knockout was performed by homologous recombination, the transformed using lithium acetate and the strains were plated on SC agar medium (2% glucose, 0.2% amino acid mixture, 0.67% yeast nitrogen base, 2% agar), and the clone was selected by selective medium and confirmed by PCR. Unless specified, the basic medium used here was SC medium; for other carbon source media, we replaced the glucose in the SC medium with other carbon sources such as fructose, galactose, glycerol, and non-carbon source (amino acid).

Plasmid construction was performed as described (Ma et al., 2016). Take the construction of pAU34M-P_*ACT*__1_-AOX1 plasmid as an example. Using the WT strain BWP17 genome as a template, amplified the ORF fragment of AOX1 with primers AOX1-5′ and AOX1-3′ (Supplementary Table S1), digested by *Xho*? and *Sma*?, and then cloned into the plasmid pAU34M (Yu et al., 2014b), obtaining the *AOX1*-overexpressing plasmid pAU34M-P_*ACT*__1_-AOX1. The constructed overexpression plasmid was digested with *Bgl*? and transformed to obtain the overexpression strain.

### *Candida albicans* Culture

The *C. albicans* was activated at 30°C in liquid YPD (2% glucose, 1% yeast extract, 2% peptone, 80 μg/ml uridine) overnight, and the cells were transferred to SC medium for experiments and cultured to mid-log phase or stationary phase.

### Nile Red Staining

Lipid droplet staining was performed as described previously (Zhang et al., 2016). Cells were cultured to the stationary phase and collected, washed three times with phosphate buffered saline (PBS), and stained with 10 μl Nile red (1 mg/ml, dissolved in acetone) for 30 min at 30°C. The cells were examined using a microplate assay (excitation wave 488 nm, emission wave 580 nm) or photographed with a fluorescence microscope (BX-53, Olympus, Japan). All the samples were taken from triplicate independent experiments.

### ATP Assay

The ATP assay was performed as described previously (Wang et al., 2018). Briefly, the *C. albicans* were collected and washed three times with PBS, vortexed 10 times, centrifuged to remove the precipitate, and then the supernatants were collected to measure ATP using an ATP assay kit (Beyotime, China). All the samples were taken from triplicate independent experiments.

### Measurement of Glucose and Ethanol Levels

The cells were cultured to the mid-log phase, collected, and centrifuged at 10,000 (g for 5 min. The supernatant was used for glucose and ethanol determination. We used the glucose oxidase method assay kit (Applygen Technologies Inc.) to determine medium levels of glucose. The determination of ethanol content was by reference to potassium dichromate-DNS colorimetry. The results were normalized to the concentration of the cells by measuring the optical density. All the samples were taken from triplicate independent experiments.

### Measurement of Pyruvic Acid Levels, GADPH, and G3PDH Enzyme Activity

The cells were cultured to the mid-log phase, collected, and centrifuged at 10,000 × *g* for 5 min. The collected cells were added to the glass beads and vortexed for disruption. After this, they were centrifuged at 10,000 × *g* for 10 min to obtain a supernatant solution for the determination of pyruvic acid content, GADPH, and G3PDH enzyme assay. The pyruvate assay kit A081 (Nanjing jiancheng) was used to measure pyruvic acid levels; GAPDH and G3PDH enzyme activity assay as reference (Saavedra et al., 2008). All the samples were taken from triplicate independent experiments.

### NADH Assay

NADH assay was performed as described previously (He et al., 2013). The strains were cultured to the mid-log phase, and the cells were washed three times with PBS. Next, the cell lysis buffer from the NAD^+^/NADH kit was added to glass beads and vortexed several times, centrifuged at 10,000 × *g* for 10 min at 4°C, and then the supernatant was used for NAD+/NADH determination. The NAD+/NADH quantification kit was from Beyotime Biotechnology. All the samples were taken from triplicate independent experiments.

### Measurement of Mitochondrial Membrane Potential

Mitochondrial membrane potential measurement was performed as described previously (Wang et al., 2018). Briefly, the cells were collected and washed once with PBS, resuspended in 1 ml of PBS buffer, and stained with 1 μl of JC-1 [1 mg/ml, dissolved in dimethyl sulfoxide (DMSO), Sigma, United States] for 30 min at 37°C. The fluorescence of the cells was examined using a flow cytometer (CaLibar, Becton Dickinson, United States). The percentages of cells with decreased MMP (decreased fluorescence intensity in FL2) were recorded [Ex = 488 nm, FL1 (Em = 525 ± 20 nm), FL2 (Em = 585 ± 20 nm)]. All the samples were taken from triplicate independent experiments.

### 3-(4,5-Dimethylthiazol-2-yl)-2,5-Diphenyltetrazolium Bromide Assay

The MTT assay was performed as described previously (Dong et al., 2015). The overnight activated strain was transferred to the mid-log phase. Next, 1 ml of the cells was collected and washed three times with PBS, resuspended in 500 μl of MTT (100 μg/ml, diluted in different carbon sources of media), incubated at 37°C for 1 h. The pellet was resuspended in 1 ml DMSO and incubated for 5 min at 30°C. The supernatant was collected using centrifugation and measured by OD570. All the samples were taken from triplicate independent experiments.

### Measurement of Reactive Oxygen Species Levels

The ROS levels were determined as described previously (Kobayashi et al., 2002). 2′,7′-Dichlorodihydro-fluorescein diacetate (DCFH-DA, Molecular Probes, United States) dye was used to determine ROS levels. The cells were resuspended in PBS, and the final concentration of DCFH-DA (20 μg/ml) was incubated at 37°C for 30 min. The fluorescence of the cells was determined by the excitation wavelength of 488 nm and an emission wavelength of 520 nm in a fluorescence plate reader. The results were normalized to the concentration of the cell by measuring the optical density. All the samples were taken from triplicate independent experiments.

### Mitochondrial Aconitase In-Gel Enzyme Activity Assays

Protein samples were separated using a non-denaturing gel, and then the gel was incubated in a coloring solution (100 mM Tris-HCl, pH 8.0, 1 mM NADP^+^, 2.5 mM sodium aconitate, 5 mM MgCl_2_, 1.2 mM MTT, 0.3 mM phenazine methosulfate, and 5 U/ml isocitrate dehydrogenase). After the color has developed, pictures were taken using a gel imager. The determination of the activity of mitochondrial-aconitase was by comparing the activity of cytoplasm-aconitase (Pereira et al., 2019). All the samples were taken from triplicate independent experiments.

### Cell Wall Composition Measurement

β-1,3-Glucan, phosphomannan, and chitin content measurements were performed as described previously (Jia et al., 2018). Briefly, to measure chitin content, the cells were collected from the stationary phase and washed once with PBS. The cells were then resuspended in PBS, stained with CFW (with a final concentration of 100 mg/L, Sigma, United States) for 10 min, and washed twice with PBS and then the fluorescence density of the cells (excitation wave 325 nm, emission wave 435 nm) was determined using a fluorescence microplate reader (Enspire, PerkinElmer, United States). All the samples were taken from triplicate independent experiments.

### Statistical Analysis

Each experiment was performed with three replicates under the tested conditions, and the values represent the means ± standard deviations of three experiments. The difference between the strains was compared using a one-tailed Student’s *t* test or one-way ANOVA. ^∗^*P*-values < 0.05 were considered statistically significant. All statistical tests were performed using GraphPad Prism 5.

## Data Availability Statement

The original contributions presented in the study are included in the article/Supplementary Materials, further inquiries can be directed to the corresponding author/s.

## Author Contributions

XP and ML: conceptualization. XP: data curation, formal analysis, visualization, writing – original draft, writing – review and editing. ML: funding acquisition and supervision. XP, QY, TM, and YL: investigation and validation. XP and ML: methodology and project administration. All authors contributed to the article and approved the submitted version.

## Conflict of Interest

The authors declare that the research was conducted in the absence of any commercial or financial relationships that could be construed as a potential conflict of interest.
