# Supplementary material for: Study on the Function of the Inositol Polyphosphate Kinases Kcs1 and Vip1 of Candida albicans in Energy Metabolism
Source: Front Microbiol. 2020 Dec 10;11:566069. doi: 10.3389/fmicb.2020.566069 (PMC7758236; doi:10.3389/fmicb.2020.566069)
Supplement: Supplementary file 1 [file Data_Sheet_1.pdf]

Supplementary Material

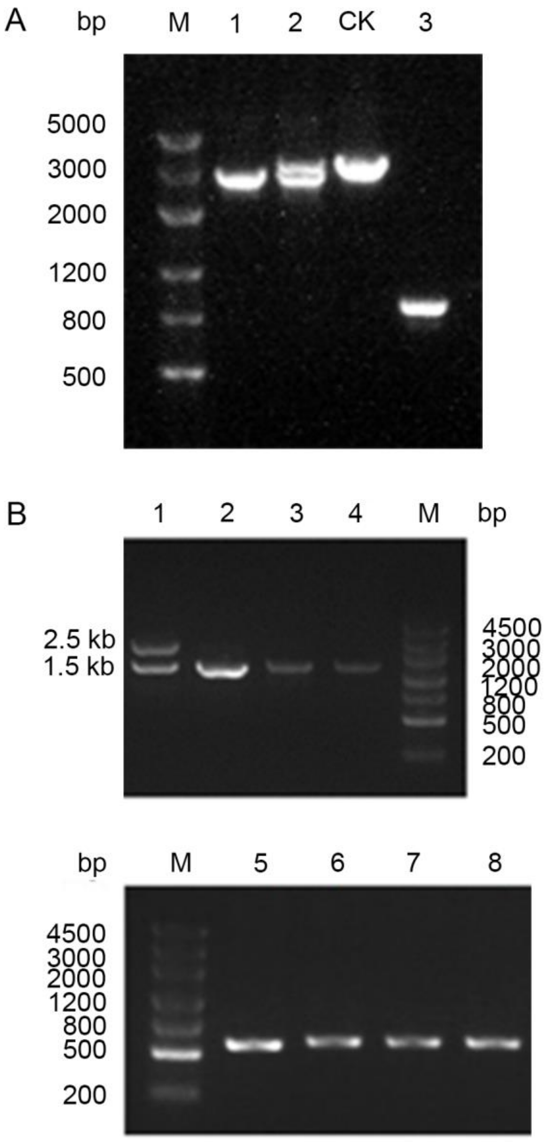

Figure S1

Construction of *vip1*Δ/Δ, *kcs1*Δ/Δ strains. A, *vip1*Δ/Δ strain construction. The primers VIP1-5det and VIP1-3det were used for PCR detection to identify heterozygous mutant strains (*vip1*::*ARG4*/*VIP1*). The No. 2 transformant showed a wild-type band of 3200 bp and an *ARG* band of 2800 bp, indicating that it was a correct transformant and the heterozygous *vip1*Δ/*VIP1* was successfully constructed.

The homozygous mutant strains (*vip1*::*ARG4*/ *vip1*:: *URA3*) were identified by PCR with VIP1-5det and VIP1-3det primers. Only the *ARG4* band of 2800 bp was shown in the No. 1 transformant, and there was no wild-type band of 3200 bp. Using VIP1-5det and URA3-3inner primers, the 800 bp *URA3* band of the No. 1 transformant could be detected (No. 3), indicating that the double copy of *VIP1* was successfully knocked out, and the homozygous mutant strain *vip1*Δ/Δ (*vip1*::*ARG4*/*vip1*::*URA3*) was successfully constructed. CK: detection primers VIP1-5det and VIP1-3det with wild-type strain genome as template, PCR to obtain 3200bp wild-type band. B, *kcs1*Δ/Δ strain construction. First, the *ARG4* marker was used to knock out a copy of *KCS1*, and the *KCS1* detection primers KCS1-5det and KCS1-3det were used to verify the transformants by PCR. It was expected that the 1.5 kb *KCS1* band and the 2.5 kb *ARG4* band will be obtained. The results of electrophoresis in the figure shown that transformant No. 1 was the correct heterozygous strain (*kcs1*::*ARG4*/*KCS1*). The PCR verification map of *kcs1*Δ/Δ homozygotes was based on heterozygotes, using URA3 markers to knock out another copy of *KCS1*. The *KCS1* detection primer KCS1-5det and the internal primer URA inner were used to verify the transformants by PCR. It was expected that a 650 bp band obtained. Figure shown that the No. 5, 6, 7, and 8 are correct homozygous strain *kcs1*Δ/Δ.

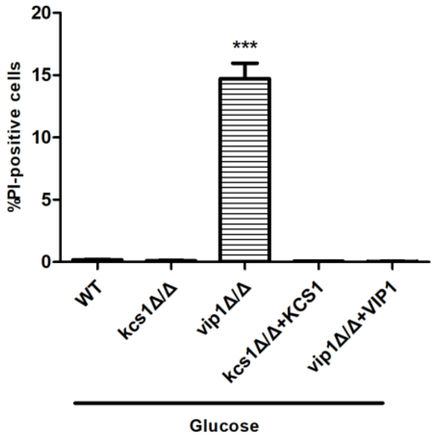

Figure S2

Increased cell membrane permeability of *vip1*Δ/Δ strains grown in glucose medium. The strains were grown in glucose medium, collected cells, PI stained, and determined by flow cytometry (BD FACSCalibur, BD, USA).

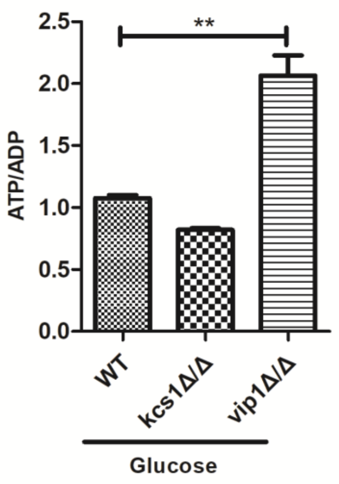

Figure S3. ATP/ADP were calculated.

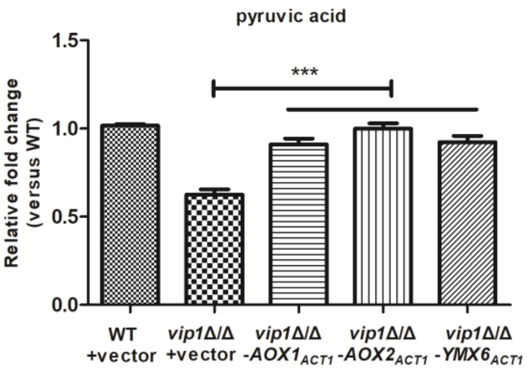

Figure S4. Determination of pyruvate content of strains grown in glucose medium.

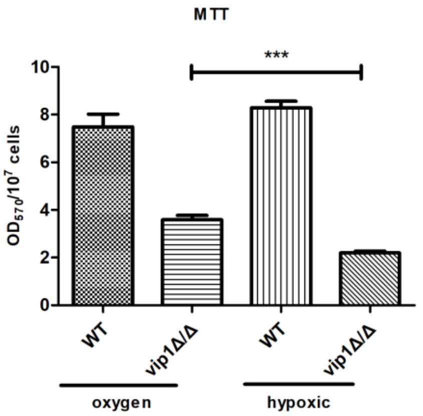

Figure S5

The *vip1*Δ/Δ strain mitochondrial succinate dehydrogenase activity decreased under hypoxic conditions. The strain was grown under aerobic or hypoxic conditions, MTT assay was used to determine the succinate dehydrogenase activity.

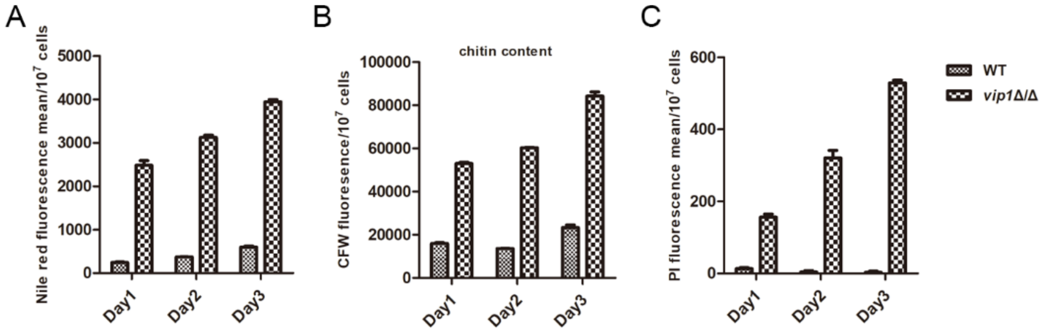

Figure S6. Determination of lipid droplets, chitin, and cell membrane permeability of strains grown in glucose medium.

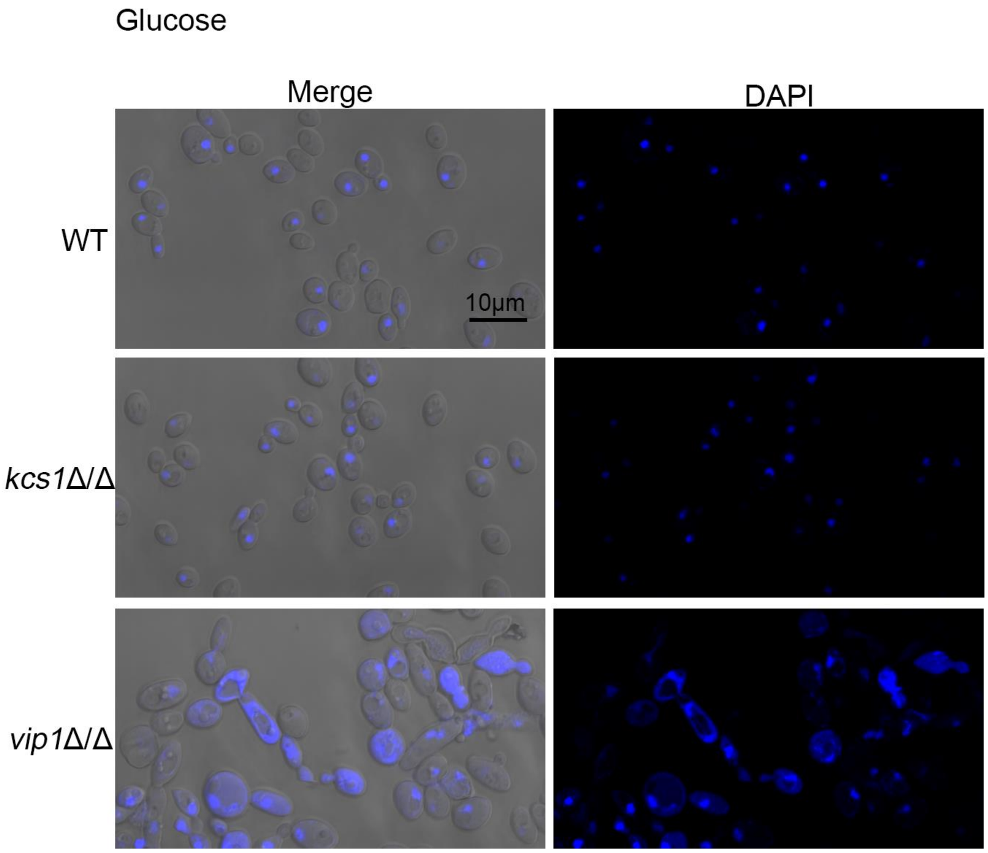

Figure S7. The nucleus morphology of the strain grown in glucose medium. The WT, *kcs1*Δ/Δ, and *vip1*Δ/Δ strains were grown in glucose medium, and the cells were collected and stained with DAPI to observe the nucleus morphology.

Table S1.

Description	Name	Sequence
KCS1 deletion,5' flank	KCS1-5DR	AAACTTGTGGGAGATATTTCCATCTATTTTTTTTTAGTCACATTTGC ACGATTTCTTACGTTTCCCAGTCACGACGTT
KCS1 deletion,3' flank	KCS1-3DR	GCACATCTATACACACACACACATACACACAGAGTATTTCTCTGAC TCGTTTATTTACTTTGGAATTGTGAGCGGATA
KCS1 detection,5' flank	KCS1-5det	ACCATCCCTTTATCATCCCATC
KCS1 detection,3' flank	KCS1-3det	CTTGTTTGCTACCATATGATCAT
KCS1 complementation,5' flank	KCS1-5C	GAACACCACGTTTTGCAATTTCCA
KCS1 complementation,3' flank	KCS1-3C	ACAGAGTATTTCTCTGACTCGT
VIP1 deletion,5' flank	VIP1-5DR	AATTAGCAGAGACGGCGGGCCTCATTTGGATTCGCAGTTATTGGA AAGATTAAAAGAAATTTTCCCAGTCACGACGTT
VIP1 deletion,3' flank	VIP1-3DR	ATCGGAAAAAATCAAGAGTATTTTAGATAACTGGGAACAATACGAA TAAACTTAGAATGTTGGAATTGTGAGCGGATA
VIP1 detection,5' flank	VIP1-5det	TGTCGATGGGGCAAGAATC
VIP1 detection,3' flank	VIP1-3det	AAAATCGTCCGTCAAGCCT
VIP1 complementation,5' flank	VIP1-5C	CCGGAATTCCGGAGAGTATTTTAGATAACTGGG
VIP1 complementation,3' flank	VIP1-3C	TCCCCCGGGGGACCAAAACACATGCAGAAGAT
AOX1 plasmid construction, 5' flank	AOX1-5'	CCGCTCGAG ATGATTGGTTTATCTACTTATAGAA
AOX1 plasmid construction, 3' flank	AOX1-3'	TCCCCCGGGAAGATACAAATCCTTTCTTTCC
AOX2 plasmid construction, 5' flank	AOX2-5'	CCGCTCGAG ATGCTTACTGCTTCGCTTTACA
AOX2 plasmid construction, 3' flank	AOX2-3'	TCCCCCGGG TAATTGTAAATCTTGTTTTTCCC
YMX6 plasmid construction, 5' flank	YMX6-5'	CCGCTCGAG ATGCGCAGTGCGATAAAG
YMX6 plasmid construction, 3' flank	YMX6-3'	TCCCCCGGGCTCTCTAGAACAATCTCTAC

Table S1. Primers

Table S2.

Strains	Genotype	Sources
BWP17(wild-type/WT)	*ura3Δ::λimm434/ura3Δ::λimm434 his1::hisG/his1::hisG arg4::hisG/arg4::hisG*	Dana A.Davis
kcs1Δ/Δ	*ura3Δ::λimm434/ura3Δ::λimm434 his1::hisG/his1::hisG arg4::hisG/arg4::hisG kcs1::ARG4/kcs1:: dpl200*	Qilin Yu
vip1Δ/Δ	*ura3Δ::λimm434/ura3Δ::λimm434 his1::hisG/his1::hisG arg4::hisG/arg4::hisG vip1::ARG4/vip1:: dpl200*	Tianyu Ma
kcs1Δ/Δ + KCS1	*ura3Δ::λimm434/ura3Δ::λimm434 his1::hisG/his1::hisG arg4::hisG/arg4::hisG kcs1::ARG4/kcs1:: dpl200, pDDB78-KCS1*	Qilin Yu
vip1Δ/Δ + VIP1	*ura3Δ::λimm434/ura3Δ::λimm434 his1::hisG/his1::hisG arg4::hisG/arg4::hisG vip1::ARG4/vip1:: dpl200, pDDB78-VIP1*	This study
vip1Δ/Δ + AOX1	*ura3Δ::λimm434/ura3Δ::λimm434 his1::hisG/his1::hisG arg4::hisG/arg4::hisG vip1::ARG4/vip1:: dpl200, pACT1-AOX1*	This study
vip1Δ/Δ + AOX2	*ura3Δ::λimm434/ura3Δ::λimm434 his1::hisG/his1::hisG arg4::hisG/arg4::hisG vip1::ARG4/vip1:: dpl200, pACT1-AOX2*	This study
vip1Δ/Δ + YMX6	*ura3Δ::λimm434/ura3Δ::λimm434 his1::hisG/his1::hisG arg4::hisG/arg4::hisG vip1::ARG4/vip1:: dpl200, pACT1-YMX6*	This study
WT + Vector	*ura3Δ::λimm434/ura3Δ::λimm434 his1::hisG/his1::hisG arg4::hisG/arg4::hisG, pACT1*	This study
vip1Δ/Δ + Vector	*ura3Δ::λimm434/ura3Δ::λimm434 his1::hisG/his1::hisG arg4::hisG/arg4::hisG vip1::ARG4/vip1:: dpl200, pACT1*	This study

Table S2. *C. albicans* strains
